# Successful treatment of hemoptysis caused by a type 2 endoleak after thoracic endovascular aortic repair

**DOI:** 10.1186/s42155-018-0019-z

**Published:** 2018-10-22

**Authors:** Eijun Sueyoshi, Hirofumi Koike, Ichiro Sakamoto, Masataka Uetani

**Affiliations:** 1 Department of Radiology, Nagasaki University School of Medicine, Nagasaki, Japan; 2 Department of Radiological Science, Nagasaki University Graduate School of Biomedical Sciences, Nagasaki, Japan

**Keywords:** Aortic aneurysm, Rupture, Hemoptysis, Embolization, Endoleak

## Abstract

**Background:**

Massive hemoptysis is a life-threatening condition and can arise as a complication of conditions. Conversely, hemoptysis rarely occurs as a complication of a ruptured thoracic aortic aneurysm (TAA).

**Case presentation:**

A 76-year-old male had a history of surgical replacement of the whole aortic arch due to a TAA. Three years after the surgery, severe hemoptysis occurred, which resulted in the patient’s emergency hospitalization at our hospital. The patient was diagnosed with ruptured pseudoaneurysms of the aortic arch. Emergency thoracic endovascular aortic repair (TEVAR) was performed. .

After that, the hemoptysis stopped, and the patient was discharged. Two months later, the hemoptysis reccurred so the patient was re-admitted to our hospital. CT showed a type 2 endoleak from the bronchial artery. The anastomotic pseudoaneurysms remained. As re-rupturing of the anastomotic aneurysms due to a type 2 endoleak was suspected, transcatheter arterial embolization was performed to treat the type 2 endoleak. The patient’s hemoptysis stopped, and he was discharged.

One year later, CT showed that the anastomotic pseudoaneurysms had disappeared, and the diameter of the aorta had also reduced.

**Conclusion:**

We present a case of hemoptysis caused by a type 2 endoleak that occurred after TEVAR for a ruptured TAA. The hemoptysis was secondary to aortobronchial fistulas caused by anastomotic aortic pseudoaneurysms. Transcatheter arterial embolization of the type 2 endoleak was very effective against the hemoptysis, and the pseudoaneurysms also disappeared. No such cases have been reported previously.

## Background

Massive hemoptysis is a life-threatening condition and can arise as a complication of conditions such as tuberculosis, bronchiectasis, bronchial carcinoma, and pulmonary aspergilloma (Julià-Serdà et al. 1996). Conversely, hemoptysis rarely occurs as a complication of a ruptured thoracic aortic aneurysm (TAA). However, in a large percentage of cases of ruptured TAA-induced hemoptysis the hemoptysis is massive, and hence, such cases are associated with a high mortality rate when they are not diagnosed and treated early (Julià-Serdà et al. 1996). Recently, thoracic endovascular aortic repair (TEVAR) has become a common treatment for ruptured TAA.

We present a case of hemoptysis secondary to aortobronchial (AB) fistula formation caused by a type 2 endoleak, which arose after TEVAR for a ruptured TAA.

## Case presentation

A 76-year-old male had a history of surgical replacement of the whole aortic arch due to a TAA (total arch replacement with elephant trunk). Three years after the surgery, severe hemoptysis occurred, which resulted in the patient’s emergency hospitalization at our hospital. On arrival, massive hemoptysis (400 mL) and hypotension were seen. The patient’s initial blood pressure was 79/42 mmHg. His hypotension resolved after the intravenous administration of crystalloid fluids. Laboratory tests showed a hemoglobin level of 7.3 g/dL. The patient received blood transfusions, and a computed tomography (CT) scan was performed.

**Fig. 1:**
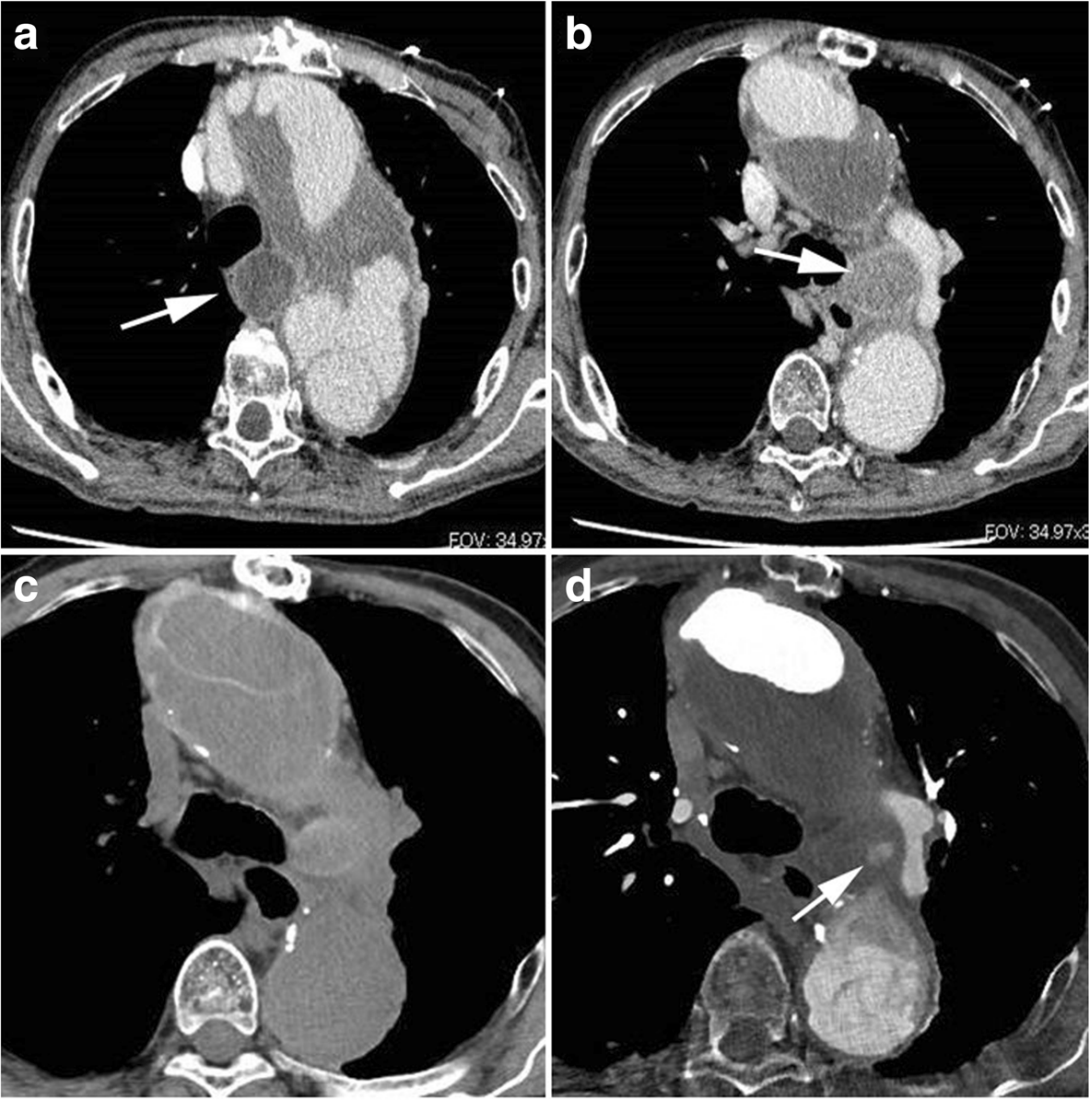
A 76-year-old male with massive hemoptysis. **a** and **b**. CT showed two aortic pseudoaneurysms (arrows), which were in contact with the aortic anastomoses. They were attached to the trachea and left bronchus, respectively, which was suggestive of AB fistula formation. **c** and **d**. Two months before **a** and **b**, CT showed that an aortic pseudoaneurysm had blood flow (arrow). After that, the psuedoaneurysn increased in size

The CT images showed two aortic pseudoaneurysms, related to the aortic anastomoses. They were attached to the trachea and the left bronchus, which was suggestive of AB fistula formation (Fig. 1). The patient was diagnosed with ruptured pseudoaneurysms of the aortic arch. Emergency TEVAR was performed under general anethesia. Two conformable TAG thoracic devices (W.L Gore and Associates, Flagstaff, AZ, USA; diameter: 34 mm x length: 200 mm, diameter: 37 mm x length: 200 mm) were inserted into the region extending from the aortic arch (elephant trunk) to the descending aorta.

**Fig. 2:**
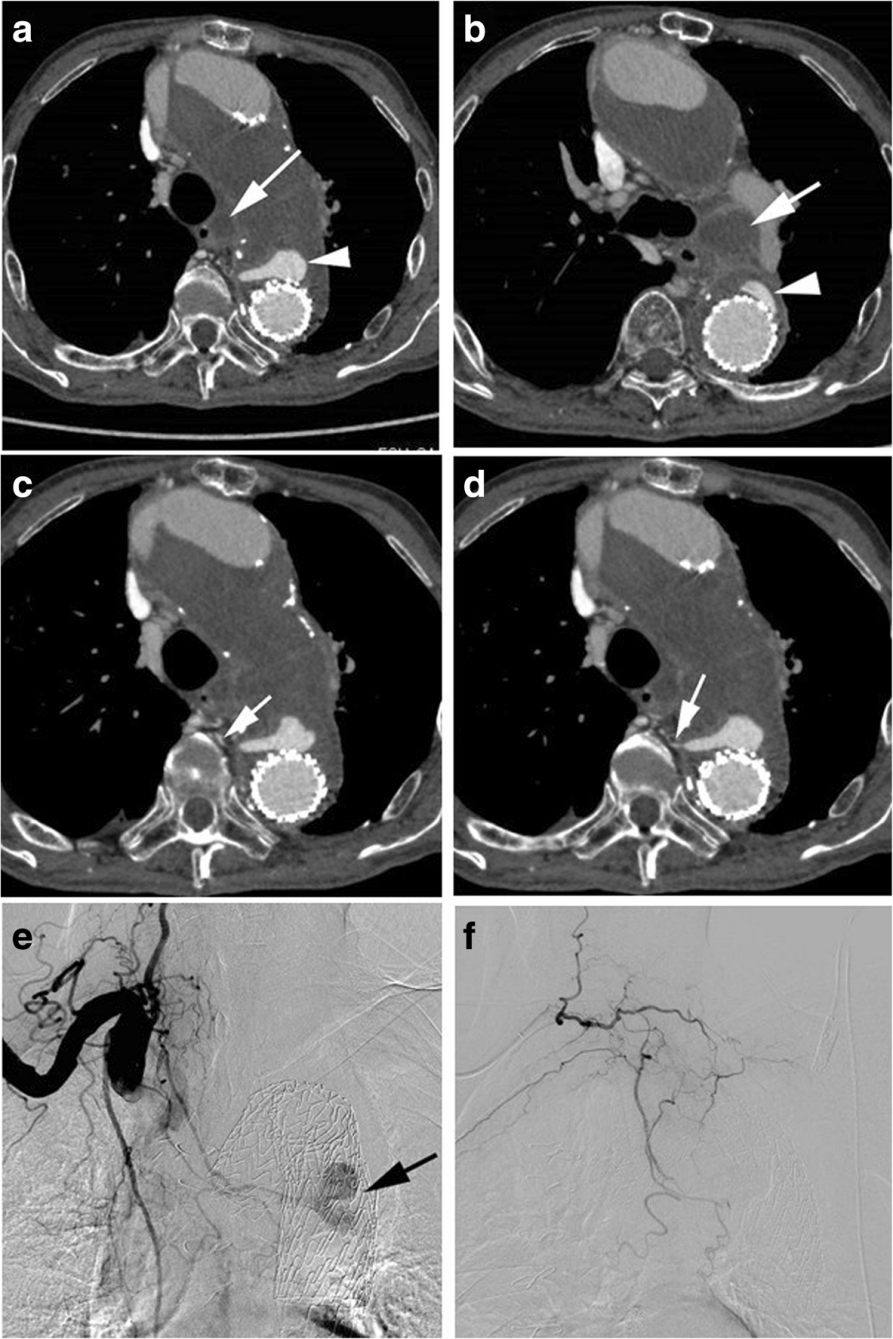
**a** and **b**. Two stent-grafts were inserted into the region extending from the aortic arch (elephant trunk) to the descending aorta. CT showed a type 2 endoleak. The pseudoaneurysms remained (arrows). **c** and **d**. CT showed a type 2 endoleak via the bronchial artery (arrows). **e**. Brachiocephalic arteriography showed a type 2 endoleak (arrow) into the aortic pseudoaneurysms from the supreme intercostal artery via the bronchial artery. **f**. After TAE, the type 2 endoleak disappeared

After that, the hemoptysis stopped, and the patient was discharged. Two months later, the hemoptysis reccurred so te patient was re-admitted to our hospital. CT showed a type 2 endoleak from the bronchial artery. The pseudoaneurysms remained (Figs. 2a and b). At that time, blood test results were as follows: hemoglobin level of 9.3 g/dL; peripheral white blood cells 5.4 × 10^9^/L; C-reactive protein 0.4 mg/L; and erythrocyte sedimentation rate 12 mm/h.

As re-rupturing of the pseudoaneurysms due to a type 2 endoleak was suspected, transcatheter arterial embolization (TAE) was performed to treat the type 2 endoleak.

First, the femoral artery was punctured, and aortography was conducted. Aortography did not show any type 1 or 3 endoleaks. However, a type 2 endoleak from the brachiocephalic artery was confirmed.

**Fig. 3:**
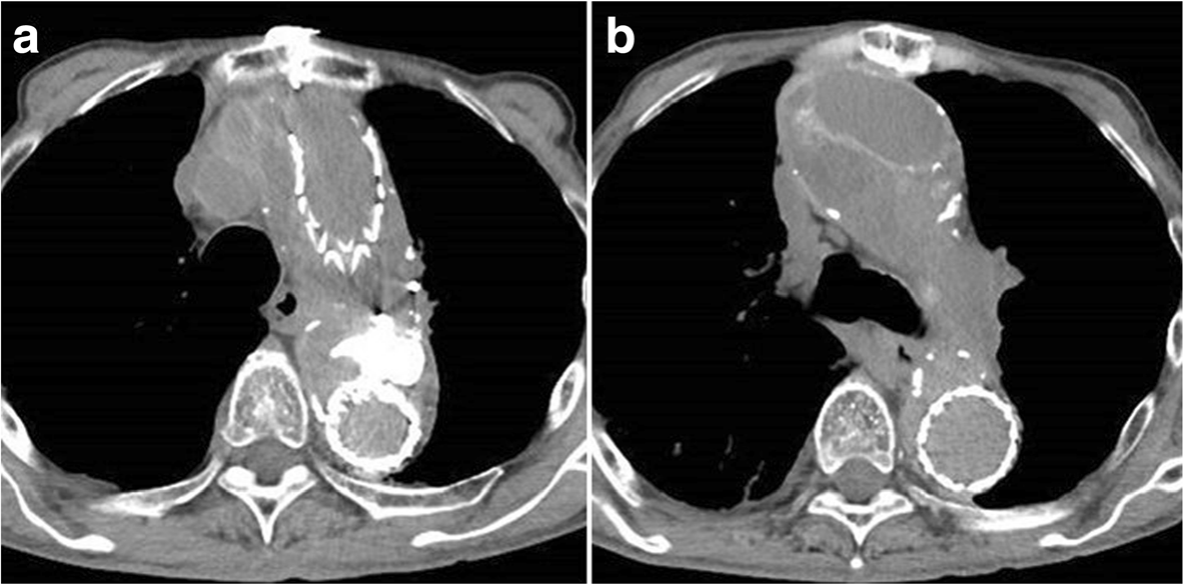
**a** and **b**. One year later, CT showed that the anastomotic pseudoaneurysms had disappeared, and the diameter of the aorta had also reduced

Next, the right brachial artery was punctured, and brachiocephalic arteriography was carried out, which showed a type 2 endoleak into the aortic pseudoaneurysms from the supreme intercostal artery via the bronchial artery (Fig. 2c). We used the triple coaxial technique to treat it. A 4F diagnostic catheter was advanced through a 2.7-F non-tapered microcatheter (Sniper 2 Selective Revolution; Terumo-Clinical Supply). Next, a 1.9-F non-tapered microcatheter (Marvel; Tokai, Kasugai, Japan) was advanced through a 2.7-F microcatheter, which was coaxially introduced through a catheter. The 1.9-F non-tapered microcatheter was advanced into the aortic sac via the bronchial artery. TAE was performed by injecting 5 ml of an *n*-butyl cyanoacrylate (NBCA) (B. Braun, Melsungen, Germany): Lipiodol Ultra-Fluide (Guerbet, Roissy, France) (ratio: 1: 3) mixture into the aortic sac and pseudoaneurysms via the inflowing artery. After the TAE, the type 2 endoleak disappeared (Fig. 2d). The patient’s hemoptysis stopped, and he was discharged. There was no further recurrence of the hemoptysis. One year later, CT showed that the pseudoaneurysms had disappeared, and the diameter of the aorta had also reduced (Fig. 3). Gallium scintigraphy showed no abnormal accumulation. The patient’s progress has been good.

Hemoptysis is a rare complication of TAA. It usually occurs after the formation of an AB fistula. In such cases, the hemoptysis can be massive if it is not treated surgically (Ahmadi et al. 2014). A high degree of suspicion regarding this entity is the key to its appropriate management. It is estimated that 5% of patients who present with hemoptysis suffer massive hemoptysis, a life-threatening condition that can cause airway obstruction and exsanguination. It exhibits a mortality rate of 30–70% (Phang et al. 2001). Nowadays, such hemoptysis can be treated using endovascular techniques involving stent-grafts (Kokotsakis et al. 2007).

In this case, we first treated the AB fistula formed by the rupturing of the anastomotic pseudoaneurysms using TEVAR. The hemoptysis initially stopped because the TEVAR blocked the blood flow to the pseudoaneurysms and reduced the pressure within them. However, 2 months later the hemoptysis relapsed because a type 2 endoleak increased the pressure within the aortic sac and pseudoaneurysms. Moreover, the type 2 endoleak supplied blood flow into the aortic sac and pseudoaneurysms.

Previously, Synowiec T, et al. reported that hemoptysis was a unique symptom of type 1A endoleaks that occur after TEVAR and led to the rupturing of thoracic aneurysms (Synowiec et al. 2013). However, to the best of our knowledge, this is the first report about hemoptysis caused by a type 2 endoleak after TEVAR.

The incidence of endoleak after TEVAR ranges from 5% to 20%, which is similar to that after endovascular abdominal aortic aneurysm repair (EVAR) (Parmer et al. 2006). Type 2 endoleaks are most commonly seen after EVAR. Type 1 and 2 endoleaks occur at similar rates after TEVAR (Stavropoulos et al. 2009). An accepted management method is aggressive endovascular repair of type 1 and 3 endoleaks along with observation for type 2endoleaks (Alsac et al. 2011). Collateral circulation in the chest involving the thoracic aorta is not so well developed compared to collateral vessels in the abdomen, making transarterial embolization of thoracic endoleaks difficult (Stavropoulos et al. 2009). To our knowledge, there is no consensus treatment option for type 2 endoleaks.

In the present case, rupturing and hemoptysis occurred due to a type 2 endoleak, which was considered to be an absolute indication for embolization.

It is still unclear if embolization of the endoleak nidus alone achieves the same outcomes as embolization of the endoleak nidus and the associated branch vessels. In the current case, using the triple coaxial technique, a microcatheter was inserted into the aortic sac via the bronchial artery. TAE was performed by injecting 5 ml of an A: Lipiodol Ultra-Fluide (ratio: 1: 3) mixture into the aortic sac/pseudoaneurysms via the inflowing artery. After the TAE, the type 2 endoleak disappeared. In this case, TAE was very effective against the patient’s hemoptysis, and the pseudoaneurysms disappeared. However, further investigation is necessary to identify more effective and appropriate embolization methods.

## Conclusions

We described a case of hemoptysis secondary to AB fistula formation, which was caused by a type 2 endoleak that developed after TEVAR for a ruptured TAA. No such cases have been reported previously. TAE of the type 2 endoleak was very effective against the hemoptysis, and the pseudoaneurysms also disappeared.

## Abbreviations

AB fistula: Aortobronchial fistula
CT: Computed tomography
NBCA: *N*-butyl cyanoacrylate
TAA: Thoracic aortic aneurysm
TAE: Transcatheter arterial embolization
TEVAR: Thoracic endovascular aortic repair
